# Surviving rectal cancer at the cost of a colostomy: global survey of long-term health-related quality of life in 10 countries

**DOI:** 10.1093/bjsopen/zrac085

**Published:** 2022-12-21

**Authors:** Helle Ø Kristensen, Anne Thyø, Katrine J Emmertsen, Neil J Smart, Thomas Pinkney, Andrea M Warwick, Dong Pang, Hossam Elfeki, Mostafa Shalaby, Sameh H Emile, Mohamed Abdelkhalek, Mohammad Zuhdy, Tomas Poskus, Audrius Dulskas, Nir Horesh, Edgar J B Furnée, Sanne J Verkuijl, Nuno José Rama, Hugo Domingos, João Maciel, Alejandro Solis-Peña, Eloy Espín-Basany, Marta Hidalgo-Pujol, Sebastiano Biondo, Annika Sjövall, Peter Christensen

**Affiliations:** Department of Surgery, Aarhus University Hospital, Aarhus, Denmark; Danish Cancer Society Centre for Research on Survivorship and Late Adverse Effects After Cancer in the Pelvic Organs, Aarhus, Denmark; Department of Surgery, Aarhus University Hospital, Aarhus, Denmark; Danish Cancer Society Centre for Research on Survivorship and Late Adverse Effects After Cancer in the Pelvic Organs, Aarhus, Denmark; Surgical Department, Randers Regional Hospital, Randers, Denmark; Department of Surgery, Aarhus University Hospital, Aarhus, Denmark; Danish Cancer Society Centre for Research on Survivorship and Late Adverse Effects After Cancer in the Pelvic Organs, Aarhus, Denmark; Surgical Department, Randers Regional Hospital, Randers, Denmark; Royal Devon and Exeter NHS Foundation Trust, Royal Devon and Exeter Hospital, Exeter, UK; University Hospitals Birmingham NHS Foundation Trust, Birmingham, UK; Brisbane Academic Functional Colorectal Unit, QEII Hospital, Brisbane, Queensland, Australia; Peking University School of Nursing, Peking, China; Colorectal Surgery Unit, Mansoura University Hospital, Mansoura, Egypt; Colorectal Surgery Unit, Mansoura University Hospital, Mansoura, Egypt; Colorectal Surgery Unit, Mansoura University Hospital, Mansoura, Egypt; Surgical Oncology Department, Oncology Center Mansoura University (OCMU), Mansoura, Egypt; Surgical Oncology Department, Oncology Center Mansoura University (OCMU), Mansoura, Egypt; Department of Abdominal and General Surgery and Oncology, Faculty of Medicine, Vilnius University, National Cancer Institute, Vilnius, Lithuania; Department of Abdominal and General Surgery and Oncology, Faculty of Medicine, Vilnius University, National Cancer Institute, Vilnius, Lithuania; Sheba Medical Center, Ramat Gan, Israel; Department of Surgery, Division of Abdominal Surgery, University of Groningen, University Medical Center Groningen, Groningen, The Netherlands; Department of Surgery, Division of Abdominal Surgery, University of Groningen, University Medical Center Groningen, Groningen, The Netherlands; Surgery Colorectal Unit, Centro Hospitalar de Leiria, Leiria, Portugal; Colorectal Surgery Unit, Champalimaud Foundation, Lisbon, Portugal; Colorectal Surgery Unit, Instituto Português de Oncologia, Lisbon, Portugal; Colorectal Surgery Unit, General Surgery Department, Universitat Autonoma de Barcelona, Hospital Vall d’Hebron, Barcelona, Spain; Colorectal Surgery Unit, General Surgery Department, Universitat Autonoma de Barcelona, Hospital Vall d’Hebron, Barcelona, Spain; Department of General and Digestive Surgery, Colorectal Unit, Bellvitge University Hospital, University of Barcelona and IDIBELL, Barcelona, Spain; Department of General and Digestive Surgery, Colorectal Unit, Bellvitge University Hospital, University of Barcelona and IDIBELL, Barcelona, Spain; Division of Coloproctology, Department of Pelvic Cancer, Karolinska University Hospital, Stockholm, Sweden; Department of Molecular Medicine and Surgery, Karolinska Institutet, Stockhom, Sweden; Department of Surgery, Aarhus University Hospital, Aarhus, Denmark

## Abstract

**Background:**

Colorectal cancer management may require an ostomy formation; however, a stoma may negatively impact health-related quality of life (HRQoL). This study aimed to compare generic and stoma-specific HRQoL in patients with a permanent colostomy after rectal cancer across different countries.

**Method:**

A cross-sectional cohorts of patients with a colostomy after rectal cancer in Denmark, Sweden, Spain, the Netherlands, China, Portugal, Australia, Lithuania, Egypt, and Israel were invited to complete questionnaires regarding demographic and socioeconomic factors along with the Colostomy Impact (CI) score, European Organisation for Research and Treatment of Cancer (EORTC) Quality of Life Questionnaire (QLQ-C30) and five anchor questions assessing colostomy impact on HRQoL. The background characteristics of the cohorts from each country were compared and generic HRQoL was measured with the EORTC QLQ-C30 presented for the total cohort. Results were compared with normative data of reference European populations. The predictors of reduced HRQoL were investigated by multivariable logistic regression, including demographic and socioeconomic factors and stoma-related problems.

**Results:**

A total of 2557 patients were included. Response rates varied between 51–93 per cent. Mean time from stoma creation was 2.5–6.2 (range 1.1–39.2) years. A total of 25.8 per cent of patients reported that their colostomy impairs their HRQoL ‘some’/‘a lot’. This group had significantly unfavourable scores across all EORTC subscales compared with patients reporting ‘no’/‘a little’ impaired HRQoL. Generic HRQoL differed significantly between countries, but resembled the HRQoL of reference populations. Multivariable logistic regression showed that stoma dysfunction, including high CI score (OR 3.32), financial burden from the stoma (OR 1.98), unemployment (OR 2.74), being single/widowed (OR 1.35) and young age (OR 1.01 per year) predicted reduced stoma-related HRQoL.

**Conclusion:**

Overall HRQoL is preserved in patients with a colostomy after rectal cancer, but a quarter of the patients interviewed reported impaired HRQoL. Differences among several countries were reported and socioeconomic factors correlated with reduced quality of life.

## Teaser

In this global survey among 2557 individuals with a colostomy after rectal cancer, generic and stoma-specific HRQoL differed significantly between countries; however, it resembled that of country-specific population norms. The most important predictors of stoma-related reduced HRQoL were stoma dysfunction and being financially burdened by the colostomy.

## Introduction

Colorectal cancer is the most common reason for formation of an end stoma^1,2^, and it is well established that some patients have long-term reduced health-related quality of life (HRQoL) because of this condition^3^. Nevertheless, literature indicates that the impact of a stoma on HRQoL depends not only on the altered anatomy and the consequent stoma-related difficulties and complications, but it is also due to demographic^4–8^ and socioeconomic factors^9–11^ and cultural features^12^; however, previous research on the differences in HRQoL among people with a stoma of different sex, age, and socioeconomic status reported conflicting results. A few studies found poorer HRQoL in younger compared with older patients^6,11^, whereas others did not find an impact of age^8,10,13^. Studies on differences in HRQoL between sexes have also shown varying results with most reporting poorer HRQoL in women^8,10,11,14^ and others showing no differences. Furthermore, although differences between sociodemographic groups have been reported^13^, most research documented no difference in HRQoL when comparing groups with respect to education, employment status and household income. To date, most studies have been conducted on small (fewer than 100 patients) cross-sectional cohorts^10,13^ and those comparing HRQoL in patients with ostomies across geographical and cultural borders are very limited^12^. On this basis, this study aimed to investigate the predictors of self-reported stoma-related reduced HRQoL by investigating differences in generic HRQoL in individuals with a permanent colostomy after rectal cancer in 10 different countries.

## Methods

Participating centres in Denmark, Sweden, Spain, China, the Netherlands, Portugal, Australia, Egypt, Lithuania and Israel formed an ad hoc collaboration for the purpose of this study, and identified eligible patients (those with a permanent colostomy after rectal cancer) from national or regional databases, hospital record systems or in stoma clinics. Inclusion criteria were surgery with curative intent for rectal cancer with the formation of a permanent colostomy. Exclusion criteria were age under 18 years, recurrence and a time since stoma creation of less than 12 months. Participating centres were responsible for securing local approvals. Eligible patients were contacted in person, by phone or by mail depending on local setup and were invited to self-complete a web-based or pen-and-paper version of the questionnaires or invited to an interview. Non-responders in all countries received a second invitation or a phone call, except for in Israel and Australia due to local organizational circumstances, including for example, enrolment in a stoma care clinic.

Disease and treatment-specific data (ASA score, TNM stage, oncological treatment, procedure, setting, complications and time since stoma creation) were collected from a national database (Denmark, Sweden and the Netherlands) or from hospital databases or hospital charts (Spain, China, Portugal, Australia, Lithuania, Egypt and Israel).

### Questionnaires

Participants completed a questionnaire on demographic (sex, weight, height and age) and socioeconomic information (employment status, marital status, educational level and whether the stoma burdens the household finances), five anchor questions stating the overall impact of the colostomy on aspects of their lives (quality of life, satisfaction with life, adjustment, embarrassment and restrictions in everyday activities), the Colostomy Impact (CI) score^15^ and the European Organization for Research and Treatment of Cancer (EORTC) Quality of Life Questionnaire Core30 (QLQ-C30)^16^.

In brief, the CI score is a patient-reported outcome measure developed at Aarhus University Hospital assessing stoma dysfunction. It is short and simple, consisting of seven items covering stoma-related problems, symptoms and complications that are associated with HRQoL. The CI score has been validated in all participating countries^17^, except for Egypt, Lithuania and Israel, where validation is currently underway. The CI score has a weighted scoring system and provides a total sum score ranging from 0 to 38. A CI score of 0–9 points is categorized as minor CI, representing better stoma function and a score of 10–38 points is categorized as major CI, representing worse stoma function. Calculation of the CI score requires completion of all items, and patients with one or more missing items were excluded from analyses involving the CI score.

EORTC QLQ-C30 version 3.0 is a multidimensional generic HRQoL measure for patients with cancer consisting of 30 items: 28 questions with answer options on a four-point scale, ‘not at all’, ‘a little’, ‘quite a bit’ and ‘very much’ and two questions asking responders to state overall health and overall quality of life on a seven-point scale^16^. The EORTC QLQ-C30 provides five multi-item functional scales and a multi-item global health status/quality of life scale, three multi-item symptom scales and six single-item measures. The EORTC QLQ-C30 global health status was used to testing for differences between countries to reduce multiple testing and the risk of type 1 errors^18^. All scale scores were calculated by linear transformation into scales ranging from 0 to 100, according to the scoring manual. Higher scores in the functional scales represent better functioning, whereas higher scores in the symptom scores represent worse symptoms^19^. Similarly, missing data were handled according to the scoring manual. Questionnaires that were not available beforehand in all languages, were translated by professional interpreters in accordance with the WHO recommendations of translation and adaptation of instruments. This included the background questions, anchor questions and the CI score in Arabic, Lithuanian and Hebrew, as shown in *Appendix S1*.

Results were compared with normative data published in 2019 on a reference European population obtained from a 2019 survey among representative samples of the population in 11 European countries^20^.

### Statistical analysis

Descriptive statistics are presented as mean(s.d.), mean (range) or percentages. EORTC scale scores were presented as mean(s.d.)^19^ and differences between countries were calculated with the Mann–Whitney *U* test owing to the skewness of the data. Univariate logistic regression was performed on the total cohort to identify factors potentially correlated to CI score and aspects of life as measured by the anchor questions. Variables showing differences between groups with a *P* value of less than 0.25 in univariable analysis were included in the multivariable logistic regression^21^. To identify predictors of reduced HRQoL within countries, univariable followed by a multivariable regression was conducted per country if the rule of thumb of 10 events per predictor variable was not violated. A receiver operating characteristic (ROC) was calculated along with area under the curve for the multivariable logistic regression. A significance level of 0.05 was chosen. Data collection and data management was handled with Research Electronic Data Capture (REDCap) data collection tools hosted at Aarhus University^22,23^. Statistical analyses were performed with Stata 16.1 (StataCorp, College Station, Texas, USA).

## Results

### Patients and demographics

A total of 2557 patients were included in the survey. The mean time from stoma creation was 2.5–6.2 years (range 1.1–39.2). Survey details and patient characteristics are presented per country in *Table 1*. No significant differences between countries were found regarding sex and time since stoma creation. Egyptian patients were significantly younger compared with all other countries. Chinese patients had significantly lower BMI, and no Chinese patient was categorized as having complications rated as Clavien–Dindo grade I–II. The Israeli cohort had fewer patients of TNM stage 0–I and more patients of stage II compared with patients from all other countries, and more Israeli patients were operated on in the emergency setting. No Lithuanian patients were categorized with an ASA score of 3 or higher. Besides this, there were no differences in ASA score between countries. The Swedish cohort included patients undergoing abdominoperineal excision only. No other differences were present between countries regarding the surgical procedure.

**Table 1:** Patient characteristics per country

	Denmark	Sweden	Spain	The NL	China	Portugal	Australia	Lithuania	Egypt	Israel
	1583	258	207	117	110	97	95	39	36	14
**Population/inclusion method**	National register (DCCG)	National register (SCRCR)	Hospital register	Dutch surgical colorectal audit	Hospital med. record system	Hospital database	Hospital database	Hospital database	Hospital database	Hospital database
**Response rate**	74%	61%	67%	82%	93%	80%	51%			
**MOA**										
Web-based	942 (60)	0	0	43 (37)	24 (22)	0	1 (2)	0	0	1 (7)
Pen and paper	641 (40)	258 (100)	46 (23)	73 (63)	83 (78)	29 (30)	53 (94)	0	0	1 (7)
Interview	0	0	156 (77)	0	0	67 (69)	2 (4)	38 (97)	36 (100)	12 (86)
**Sex Ratio (M:F)**										
Male	985 (63)	153 (59)	136 (66)	71 (62)	73 (66)	63 (65)	67 (68)	31 (79)	18 (50)	6 (43)
Female	584 (37)	105 (41)	70 (34)	44 (38)	37 (34)	34 (35)	31 (32)	8 (21)	18 (50)	8 (57)
**BMI mean (range)**	27.0 (14–68)	25.9 (15–61)	26.9 (15–66)	27.1 (16–55)	23.5 (16–32)	26.9 (16–35)	26.9 (16–41)	28.8 (20–38)	26.5 (19–44)	26.6 (22–32)
**Age (years) mean (range)**	74.4 (30–96)	72.7 (36–91)	76.1 (47–96)	70.7 (37–94)	67.0 (31–93)	71.9 (35–97)	69.6 (35–93)	66.3 (51–89)	49.1 (22–74)	64.2 (33–95)
**Time since stoma creation (years) (range)**	6.2 (2.1–12.2)	4.1 (1.0–7.9)	6.1 (1.0–15.1)	5.7 (1.8–10.7)	4.6 (1.0–39.2)	5.6 (1.6–16.6)	4.5 (1.0–15.0)	5.4 (2.1–12.2)	4.8 (1.6–21.7)	2.5 (1.1–5.3)
**Access stoma to nurse**										
Yes	1180 (77)	190 (77)	160 (78)	101 (89)	120 (98)	77 (87)	86 (85)	35 (90)	32 (89)	10 (71)
No	47 (3)	24 (10)	32 (16)	7 (6)	3 (2)	4 (4)	10 (10)	4 (10)	4 (11)	0
Do not know	308 (20)	34 (13)	12 (6)	6 (5)	0	8 (9)	5 (5)	0	0	4 (29)
**Stage (TNM)**										
0	0	18 (7)	10 (5)	0	3 (3)	11 (11)	3 (3)	0	0	0
I	523 (39)	83 (32)	42 (21)	18 (15)	28 (28)	30 (31)	35 (39)	8 (21)	12 (39)	0
II	426 (31)	68 (26)	59 (30)	39 (34)	32 (32)	28 (29)	28 (31)	8 (21)	2 (6)	8 (80)
III	399 (29)	71 (27)	79 (40)	59 (51)	25 (25)	24 (25)	22 (24)	23 (58)	17 (55)	2 (20)
IV	5 (1)	20 (8)	8 (4)	0	12 (12)	4 (4)	2 (2)	0	0	0
**Procedure**										
Abdominal perineal excision	1237 (78)	258 (100)	167 (83)	79 (68)	105 (97)	80 (82)	70 (76)	38 (97)	29 (81)	6 (43)
Hartmann’s resection	346 (22)	0	14 (7)	21 (18)	2 (2)	9 (9)	6 (7)	1 (3)	4 (11)	7 (50)
Pelvic exenteration	0	0	5 (2)	16 (14)	0	1 (1)	15 (16)	0	3 (8)	0
**Setting**										
Acute	13 (1)	0	6 (3)	3 (3)	0	6 (6)	1 (1)	0	2 (6)	7 (50)
Elective	1570 (99)	258 (100)	195 (97)	113 (97)	95 (86)	90 (93)	87 (99)	39 (100)	34 (94)	7 (50)
**ASA score**										
1–2	1356 (86)	179 (69)	131 (66)	108 (93)	99 (80)	57 (59)	67 (77)	39 (199)	35 (97)	8 (57)
≥3	214 (14)	78 (31)	68 (34)	8 (7)	10 (8)	39 (40)	20 (23)	0	1 (3)	6 (43)
**Complications**										
No complications	1250 (79)	152 (58)	119 (59)	75 (65)	101 (83)	64 (66)	25 (26)	32 (82)	26 (72)	12 (86)
Clavien–Dindo I–II	29 (2)	71 (27)	50 (25)	21 (18)	0	16 (17)	51 (53)	4 (10)	9 (25)	1 (7)
Clavien–Dindo III	57 (4)	33 (13)	25 (12)	18 (15)	6 (5)	14 (14)	9 (9)	3(8)	1 (3)	1 (7)
Clavien–Dindo IV	19 (1)	2 (1)	6 (3)	2 (2)	0	0	2 (2)	0	0	0
Unknown	228 (14)	2 (1)	1 (1)	0	15 (12)	3 (3)	9 (9)	0	0	0

Values are *n* (%) unless otherwise indicated. Single institution. DCCG, Danish Colorectal Cancer Group; SCRCR, Swedish Colorectal Cancer Registry; MOA, mode of administration; The NL, The Netherlands.

### Quality of life

**Fig. 1:**
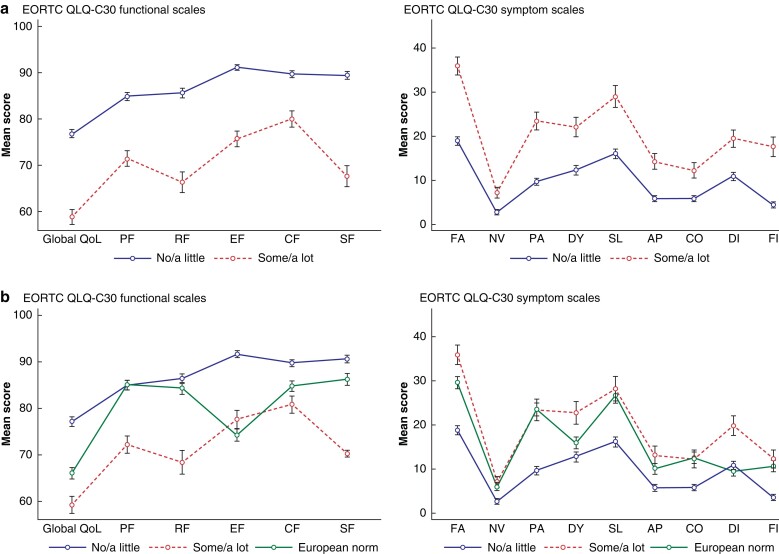
Mean scores (s.d.) of EORTC QLQ-C30 in patients reporting no/a little impaired quality of life by the colostomy *versus* patients reporting some/a lot impaired quality of life

*Figure 1* reports mean EORTC QLQ-C30 scores for the total cohort with participants divided into two groups depending on the impact of the stoma on quality of life measured by the anchor question with one group reporting ‘not at all’/‘a little’ impact on HRQoL and another group reporting ‘some’/‘a lot’ impact. Patients reporting that the colostomy impairs their quality of life ‘not at all’/‘a little’ (*n* = 1850, 74.2 per cent) had significantly higher EORTC QLQ-C30 scores across all functional scales and lower scores across all symptom scales, when compared with patients reporting that the stoma impairs their quality of life ‘some’/‘a lot’ (*n* = 642, 25.8 per cent).

**a** Total cohort. **b** European part of the cohort along with European general population normative data^17^.

EORTC QLQ-C30, European Organisation for Research and Treatment of Cancer Quality of Life Questionnaire C30; QoL, quality of life; PF, physical function; RF, role function; EF, emotional function; CF, cognitive function; SF, social function; FA, fatigue; NV, nausea/vomiting; PA, pain; DY, dyspnoea; SL, insomnia; AP, appetite loss; CO, constipation; DI, diarrhoea; FI, financial difficulties.

**Fig. 2:**
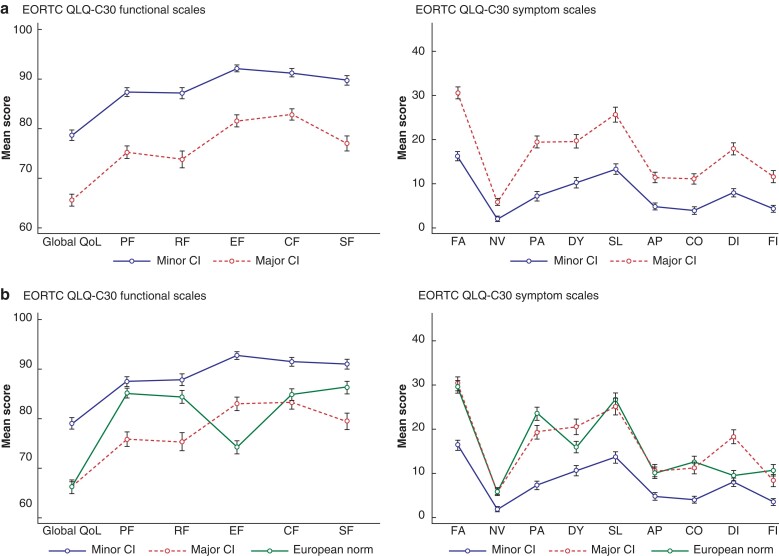
Mean scores (s.d.) of EORTC QLQ-C30 in patients with minor CI *versus* major CI

EORTC QLQ-C30 scores of the European part of the study population is seen in *Fig. 1* along with a European reference population of normative data^20^. Scale scores in the group of patients reporting ‘not at all’/‘a little’ impaired quality of life were significantly higher compared with the population norm in all functional scores, except for physical functioning and role functioning. Similarly, they had lower symptom scores in all scales except for diarrhoea. Looking at how stoma dysfunction, measured by the CI score, was related to generic HRQoL, the EORTC QLQ-C30 scores of patients with major CI and minor CI are presented in *Fig. 2*. Patients with minor CI (*n* = 1244, 51.9 per cent) had higher scores in all functional scales and lower mean scores in all symptom scales compared with patients with major CI (*n* = 1154, 48.1 per cent). When looking at the European part of the study population, patients with minor CI had significantly higher scores on all functional scales and comparable or lower in all symptom scales, except diarrhoea and dyspnoea, compared with the general European population, *Fig. 2*.

**a** Total cohort. **b** European part of the cohort along with European general population normative data^17^.

EORTC QLQ-C30, European Organisation for Research and Treatment of Cancer Quality of Life Questionnaire C30; CI, colostomy impact; QoL, quality of life; PF, physical function; RF, role function; EF, emotional function; CF, cognitive function; SF, social function; FA, fatigue; NV, nausea/vomiting; PA, pain; DY, dyspnoea; SL, insomnia; AP, appetite loss; CO, constipation; DI, diarrhoea; FI, financial difficulties.

### Differences between countries

**Fig. 3:**
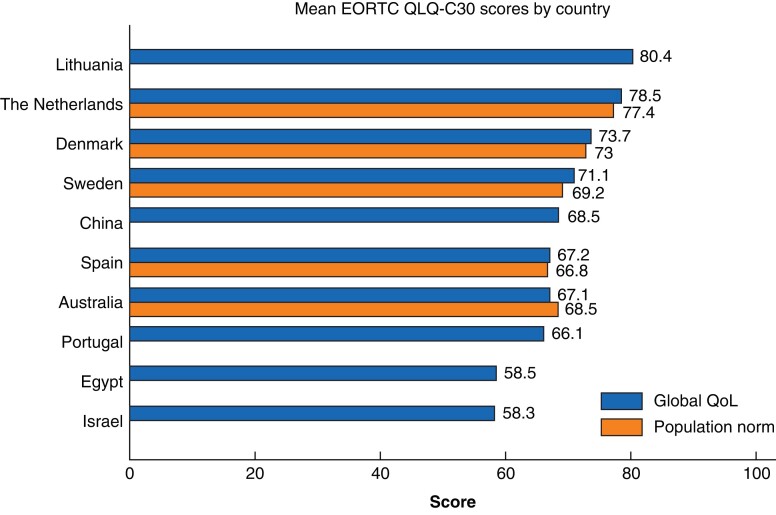
**Mean of EORTC QLQ-C30 global health status per country along with country-specific population reference values where available** ^20–22^ EORTC QLQ-C30, European Organisation for Research and Treatment of Cancer Quality of Life Questionnaire C30; QoL, quality of life.

EORTC QLQ-C30 global health status differed significantly between the countries with generally higher scores in Northern European countries. The mean global health status for each country is shown in *Fig. 3*, with reference data from the literature for the country-specific population norm where available^20,24,25^. Differences between the study cohorts of colostomy patients and general populations ranged 0.4–1.9 with a slight tendency for scores being higher in the stoma population in all countries except for Australia. Nevertheless, there were significant differences between the cohorts depending on country and mean global health status ranged from 80.4 (Lithuania) to 58.3 (Israel).

### Anchor questions

**Fig. 4:**
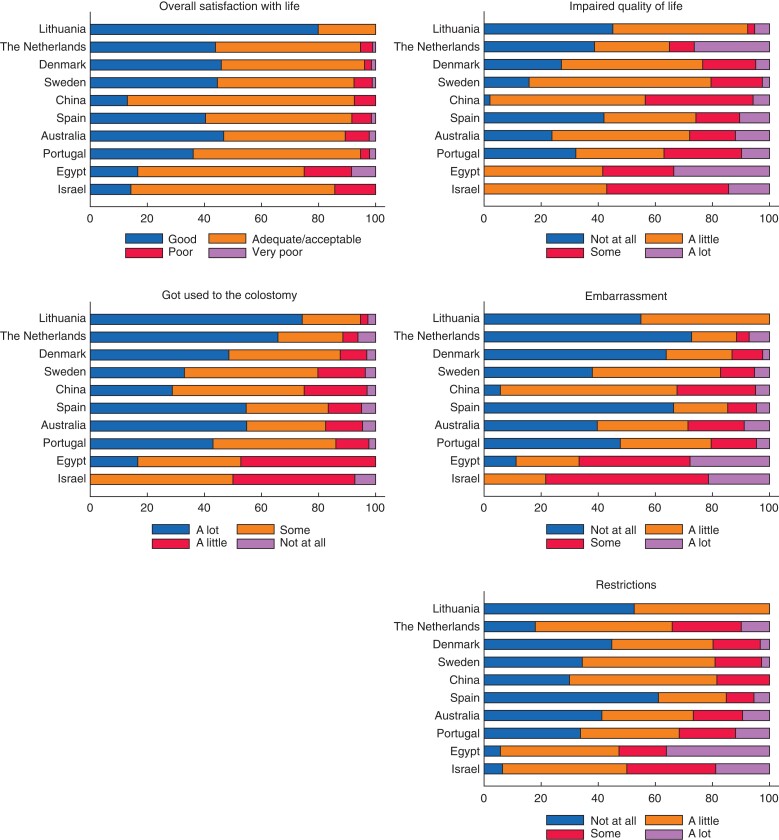
Answers to the anchor questions by country

Major variations were seen between countries in the proportions of patients reporting how their colostomy impacts different aspects of life. As seen in *Fig. 4*, patients generally reported high satisfaction with life with a colostomy with more than 80 per cent of patients stating ‘good’ or ‘adequate/acceptable’ satisfaction in all countries except for Egypt. Fewer patients in the Northern European countries reported that their stoma impairs their overall quality of life, whereas more patients in the Middle-Eastern countries and China stated that their HRQoL is impaired ‘some’/‘a lot’ by their stoma. This variance between countries was consistent throughout all anchor questions; in general, fewer Northern European patients were embarrassed or restricted and more patients had gotten used to having the stoma. In Israel and Egypt, more than half of the patients felt restricted ‘some’ or ‘a lot’ by their stoma, and 79 per cent and 67 per cent respectively were ‘some’ or ‘a lot’ embarrassed by their stoma.

### Predictors of stoma-related impaired HRQoL

Multiple logistic regression analysis was performed on the total cohort to determine predictors of self-reported reduced HRQoL, caused by the colostomy. Patients were categorized as having stoma-related reduced HRQoL if they stated ‘some’ or ‘a lot’ in the anchor question ‘Overall do you think that the colostomy impairs your quality of life?’ *Table 2* shows the predictor variables. In the multivariable analysis, stoma dysfunction measured by the CI score, young age, being single or widowed, being unemployed, and being financially burdened by the stoma were significantly associated with stoma-related reduced HRQoL. An ROC curve was calculated for the model showing area under the curve of 0.701 (95 per cent c.i. 0.675 to 0.726), *Fig. S1*.

**Table 2:** Logistic regression analysis showing correlations to the anchor question: overall, do you think that the colostomy impairs your quality of life?

	Univariable regression				Multivariable regression			
**Logistic regression**	OR	Z	*P*	95% c.i.	OR	Z	*P*	95% c.i.
**Age (years)**	1.02	−3.69	**<0.001**	1.01, 1.02	1.01	−1.97	**0**.**049**	1.00, 1.02
**Sex**	0.96	−0.41	0.684	0.79, 1.16	–	–	–	–
**BMI**	1.02	2.06	**0**.**039**	1.00, 1.04	1.00	0.35	0.723	0.98, 1.02
**Employment**								
Employed	1.00	–	–	–	–	–	–	–
Unemployed	3.17	4.79	**<0.001**	1.98, 5.09	2.74	3.52	**<0.001**	1.56, 4.82
Retired	1.05	0.40	0.689	0.82, 1.35	1.18	0.92	0.355	0.83, 1.68
**Marital status**								
Married	1.00	–	–	–	–	–	–	–
Single/widowed	1.31	2.77	**0**.**006**	1.08, 1.58	1.35	2.58	**0**.**010**	1.07, 1.69
Education*								
None	–	–	–	–	–	–	–	–
Short	0.68	−1.41	**0**.**157**	0.40, 1.15	1.08	0.25	0.800	0.57, 2.06
Long	0.64	−1.60	**0**.**109**	0.37, 1.10	0.99	−0.02	0.984	0.51, 1.93
**Financially burdened**	2.39	6.44	**<0**.**001**	1.83, 3.12	1.98	4.26	**<0**.**001**	1.44, 2.72
**Colostomy impact score**								
Minor	1.00	–	–	–	–	–	–	–
Major	3.42	12.29	**<0**.**001**	2.81, 4.15	3.32	10.69	**<0**.**001**	2.66, 4.13
**Time since stoma creation**	0.95	−2.97	**0**.**003**	0.92, 0.98	0.97	−1.31	0.189	0.92, 1.01
**Access to stoma nurse**								
Yes	1.00	–	–	–	–	–	–	–
No/do not know	0.86	−1.30	**0**.**194**	0.68, 1.08	0.92	−0.59	0.554	0.69, 1.21
**Clavien–Dindo**								
No complications	1.00	–	–	–	–	–	–	–
I–II	0.97	−0.17	0.866	–	0.79	−1.21	0.227	0.54, 1.16
III–IV	1.24	1.30	**0**.**194**	0.89, 1.71	1.03	0.14	0.886	0.70, 1.51
**Oncological treatment†**								
None	1.00	–	–	–	–	–	–	–
Any	1.06	0.32	0.748	0.73, 1.54	–	–	–	–

*Educational level was divided into three groups, where ‘short’ was less than a college degree or equivalent and patients reporting highest level of education college degree or further/higher or equivalent were grouped as having a ‘long’ education. †Any oncological treatment covered adjuvant or neoadjuvant chemo- and/or radiotherapy.

In four countries (Denmark, Sweden, Spain and the Netherlands), the number of included patients allowed for multivariable logistic regression per country. Only stoma dysfunction measured by the CI score was consistently associated with stoma-related reduced HRQoL in all four countries. In addition to stoma dysfunction, in Spanish patients, young age was associated with stoma-related impact on quality of life (*P* = 0.001), whereas in Danish patients, being single or widowed was associated with reduced stoma-related HRQoL (*P* = 0.024), as shown in *Tables S1–S4*.

## Discussion

This study demonstrates that about 25.8 per cent of patients reported that the stoma impaired their HRQoL some or a lot, but this impairment is across all EORTC QLQ-C30 scales. Even though, HRQoL resembles that of the background population. In accordance with these results, an American cross-sectional survey in patients with faecal stomas found HRQoL similar to the background population in some but not all domains of the Rand Medical Outcomes Study 36-Item Short-Form Health Survey (SF)-36^®^ version 2. And a Dutch study on individuals after colorectal cancer found HRQoL to be equal to a reference population in the elderly^6^. The impact that a stoma has on HRQoL may be more prevalent in certain high-risk patients and confined to a few HRQoL domains. A Cochrane review from 2012 concluded, however, that HRQoL is not superior in patients undergoing sphincter preservation compared with Hartmann’s operation or abdominoperineal excision^26^. It is well established that poor bowel function after a low anterior resection impacts HRQoL negatively and with the present results demonstrating that most patients with a permanent colostomy do not experience reduced HRQoL from their colostomy it should be considered that the stoma is not necessarily worse than poor bowel function after a low anterior resection.

A significant difference in generic HRQoL across the different included countries was found. A cross-sectional study from 2005 investigating HRQoL in individuals with a colostomy after rectal cancer across Europe and Middle-Eastern countries found a similar marked north-to-south gradient in HRQoL; however, when considering how the EORTC QLQ-C30 global health status resembles that of the general population in our present study, this gradient may not be attributed to the colostomy, but rather, reflects the quality of life in the background population.

The present data did reveal differences in how patients appreciated their colostomy between countries. The abovementioned north-to-south gradient was consistent in all the anchor questions with a higher proportion of patients reporting impaired HRQoL, dissatisfaction with life, restrictions, embarrassment and difficulty with adaptation in Israel, Egypt and China. The source of the geographical and cultural differences is not clear from this study, but it seems that generic HRQoL is not affected compared with the general population.

The multivariable regression showed that a number of variables were associated with reduced HRQoL. The most important risk factor was stoma dysfunction as measured by the CI score. Patients with major CI had an OR of 3.32 for reporting that their stoma reduces their quality of life compared with patients with a minor CI score. In the total cohort, young age was also associated with reduced HRQoL. Thus, for every year increase in patient age, the risk of reporting stoma-related reduced HRQoL was 1 per cent lower. Furthermore, being unemployed or retired, uneducated and financially burdened by the colostomy was associated with a higher risk of the stoma impairing HRQoL. Previous studies on socioeconomic factors have reported different results, most often reporting no difference in HRQoL depending on socioeconomic status^8–12^; however, a cross-sectional study from 2005 found low educational status to be associated with worse body image in individuals with a colostomy after colorectal cancer^13^. The only significant socioeconomic factor related to reduced stoma-related HRQoL in our study was being financially burdened by the colostomy with an OR of 1.98 of reporting that the stoma impairs HRQoL. In addition to the cost of appliances, a financial burden can occur as the result of lost income and so this question was designed to capture a financial burden from the stoma regardless of the reason. Similarly, a study on US veterans from 2007 reported that difficulty paying for ostomy supplies was associated with lower overall quality of life, but also emphasized that the causal relationship is uncertain^27^.

Among the strengths of this survey are a representation of several countries across three continents and the use of validated questionnaires. Most participants were recruited from national or hospital-based registers reducing the risk of selection bias, which is further reduced by the high response rates in most countries. Participants were assured anonymity and, where possible, patients self-completed the questionnaires. On the other hand, limitations include the cross-sectional design that does not allow for causal inferences and does not control for confounding, and the fact that the CI score has not been validated in 3 of 10 participating countries. Furthermore, information on non-responders was not available, which would have provided useful information on potential selection bias. The majority of patients in the cohort were from Denmark and this overrepresentation may affect results in the multivariable regression.

This international study documented that HRQoL even with a permanent colostomy is acceptable, and that most of the patients in fact have HRQoL at least equal to the background population as reported in the literature^20,24,25^. However, there are some patients with reduced HRQoL that should be identified and use of the CI score should be encouraged as the symptoms represented in the score (such as pain, skin problems, constipation/diarrhoea and frequent leakages) can be managed by expert stoma care nursing.

## Supplementary Material

zrac085_Supplementary_Data

## Data Availability

Data supporting the findings are available from the corresponding author upon reasonable request.
