# Supplementary material for: Surviving rectal cancer at the cost of a colostomy: global survey of long-term health-related quality of life in 10 countries
Source: BJS Open. 2022 Dec 21;6(6):zrac085. doi: 10.1093/bjsopen/zrac085 (PMC9772877; doi:10.1093/bjsopen/zrac085)
Supplement: zrac085_Supplementary_Data [file zrac085_supplementary_data.docx]

**Appendix S1. Questionnaire**

**Background questions**

What is your date of birth? (dd-mm-yyyy) _______

Indicate your sex: ☐ Male

☐ Female

How much do you weigh? ______ kg

How tall are you? ______ cm

Do you get help with everyday tasks such as personal care or cleaning around the home?

☐ I manage on my own

☐ I get help with some things

☐ I depend on other people helping me every day

What is your marital status

☐ Married/cohabiting

☐ Single

☐ Widowed

☐ Other

What was the highest level of education you completed?

☐ None

☐ Primary school

☐ Secondary school

☐ Training in a skill or trade

☐ Higher education/university

☐ Post-graduate degree

☐ I do not know

☐ I am still in full time education

What is your current employment status?

☐ Employed

☐ Unemployed

☐ Retired

Do you smoke tobacco/e-cigarettes

☐ Never smoker

☐ Ex-smoker (completely stopped at least 4 weeks ago)

☐ Smoker

How many cigarettes/cigars/e-cigarettes etc do you smoke per day, on average? ____

How much alcohol do you drink?

☐ I do not drink alcohol

☐ I drink less than 2 units per day

☐ I drink more than 2 units per day

What is your religious affiliation?

☐ Do not wish to disclose

☐ Buddhist

☐ Christian

☐ Hindu

☐ Jewish

☐ Muslim

☐ Not religious

☐ Other If ’other’: What is your religion or faith? _______

**Stomacare**

How long have you had your colostomy?

______ years

Who performs your everyday stoma care?

☐ I do

☐ My spouse/partner

☐ A family member

☐ Nurse/other health-care professional

☐ Other

How many times per day on average do you (or the person mentioned above) attend to your stoma appliance/bag?

_______ times per day

Have you ever been offered or introduced to irrigation for your colostomy management? (Irrigation is a way of controlling your bowel movements by flushing and emptying the colon at scheduled times).

☐ Yes

☐ No

Do you use irrigation for your colostomy management?

☐ No

☐ I have done it earlier, but have stopped

☐ Yes, occasionally

☐ Yes, regularly

If you use irrigation, how often on average?

____ times per week

Who pays for your stoma care products?

☐ I do /my household

☐ Private healthcare insurance

☐ Public healthcare/NHS

☐ Other

Do the costs for stoma care products confer a burden on your household finances?

☐ Yes

☐ No

☐ I do not know

Did you see a stoma care nurse after discharge from the hospital after having your colostomy?

☐ Yes

☐ No

☐ I do not know

Do you have the possibility to see a stoma care nurse if necessary?

☐ Yes

☐ No

☐ I do not know

Have you ever had surgery to your stoma or in relation to your stoma for any reason after having your colostomy? ☐ Yes

☐ No

☐ I do not know

What was the reason for this surgery? (Multiple answers possible)

☐ Narrowing

☐ Protruding

☐ Bulge

☐ Bowel obstruction

☐ Other

☐ I do not know

Do you have a bulge in relation to your colostomy?

☐ Yes

☐ No

☐ I do not know

Have you ever been told by a doctor you have a hernia in relation to your colostomy?

☐ Yes

☐ No

☐ I do not know

Do you believe you have a hernia in relation to your colostomy?

☐ Yes

☐ No

☐ I do not know

**Anchor questions**

How would you rate your overall satisfaction with your life with a colostomy?

☐ Good

☐ Adequate/acceptable

☐ Poor

☐ Very poor

Overall, do you think that the colostomy impairs your quality of life?

☐ Not at all

☐ A little

☐ Some

☐ A lot

Have you got used to having a colostomy?

☐ Not at all

☐ A little

☐ Some

☐ A lot

Are you embarrassed by your colostomy?

☐ Not at all

☐ A little

☐ Some

☐ A lot

Does your colostomy restrict you in your everyday activities or chores?

☐ Not at all

☐ A little

☐ Some

☐ A lot


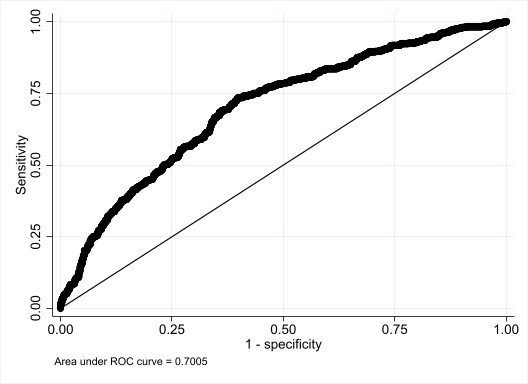


Figure S1. Receiver operating characteristic curve for the multivariable logistic regression analysis (Table 2). Area under the curve: 0.701 (95% CI: 0.675-0.726).

| DENMARK | Univariable regression | | | | Multivariable regression | | | |
| --- | --- | --- | --- | --- | --- | --- | --- | --- |
| Logistic regression | OR | Z | P | 95% CI | OR | Z | P | 95% CI |
| Age | 0.99 | -1.49 | **0.135** | 0.98-1.00 | 1.00 | 0.12 | 0.905 | 0.98-1.02 |
| Sex | 1.20 | 1.45 | **0.146** | 0.94-1.54 | 1.28 | 1.39 | 0.165 | 0.90-1.83 |
| BMI | 1.02 | 1.97 | **0.049** | 1.00-1.05 | 0.99 | -0.44 | 0.662 | 0.96-1.03 |
| Employment  Employed  Unemployed  retired | 1.00  3.54 1.07 | -  2.27  0.40 | -  **0.023**  0.688 | -  1.19-10.52  0.77-1.49 | -  2.37  1.09 | -  1.10  0.32 | **-**  0.269  0.750 | 0.51-10.97  0.63-1.90 |
| Marital status  Married  Single/widowed | 1.00  1.34 | -  2.34 | -  **0.019** | -  1.05-1.71 | -  1.51 | -  2.30 | -  **0.021** | -  1.06-2.14 |
| Education*  None  Short  long | -  0.21  0.18 | -  -1.71  -1.84 | -  **0.087**  **0.065** | -  0.03-1.25  0.03-1.11 | -  1.13  1 | -  0.67  - | -  0.501  - | -  0.79-1.62  - |
| Financially burdened | 4.69 | 3.30 | **0.001** | 1.87-11.77 | 3.43 | 1.89 | 0.058 | 0-96-12.28 |
| Colostomy Impact group  Minor CI  Major CI | 1.00  3.47 | -  9.49 | -  **<0.0001** | -  2.68-4.49 | -  3.81 | -  7.80 | -  **<0.0001** | -  2.72-5.34 |
| Time since stoma creation | .95 | -2.21 | **0.027** | 0.91-0.99 | 0.95 | -1.31 | 0.190 | 0.88-1.02 |
| Access to stoma nurse  Yes  No/do not know | 1.00  0.97 | -  -0.24 | -  0.813 | -  0.72-1.28 | - | - | - | - |
| Clavien-Dindo  No complications  I-II  III-IV | 1.00  1.05  0.74 | -  -1.00  -0.87 | -  0.319  0.385 | -  0.70-1.34  0.89-1.71 | -  -  - | -  -  - | -  -  - | -  -  - |
| Oncological treatment^¤^  None  Any | 1.00  0.83 | -  -1.33 | -  **0.185** | -  0.63-1.09 | -  0.91 | -  -0.48 | -  0.629 | -  0.63-1.33 |

Table S1: Logistic regression analysis on the Danish cohort showing correlations to the anchor question

| SWEDEN | Univariable regression | | | | Multivariable regression | | | |
| --- | --- | --- | --- | --- | --- | --- | --- | --- |
| Logistic regression | OR | Z | P | 95% CI | OR | Z | P | 95% CI |
| Age | 1.01 | 0.41 | 0.684 | 0.98-1.04 | - | - | **-** | - |
| Sex | 1.15 | 0.45 | 0.656 | 0.62-2.15 | - | - | - | - |
| BMI | 1.05 | 1.53 | **0.127** | 0.99-1.12 | 1.01 | 0.17 | 0.866 | 0.93-1.09 |
| Employment  Employed  Unemployed  retired | 1.00  2.00  0.98 | -  0.74  -0.05 | -  0.459  0.962 | -  0.32-12.51  0.45-2.13 | -  -  - | -  -  - | **-**  **-**  - | .  -  . |
| Marital status  Married  Single/widowed | 1.00  1.23 | -  0.64 | -  0.525 | -  0.65-2.36 | -  - | -  - | -  **-** | -  - |
| Education*  None  Short  long | -  1.14  1 | -  0.39  - | -  0.699  - | -  0.59-2.17  - | -  -  - | -  -  - | -  -  - | -  -  - |
| Financially burdened | 1.68 | 1.53 | **0.126** | 0.86-3.27 | 1.10 | 0.24 | 0.812 | 0.52-2.33 |
| Colostomy Impact group  Minor CI  Major CI | 1.00  5.81 | -  4.62 | -  **<0.0001** | -  2.75-12.25 | -  5.41 | -  4.02 | -  **<0.0001** | -  2.37-12.30 |
| Time since stoma creation | .90 | -1.29 | **0.198** | 0.78-1.05 | 0.93 | -0.77 | 0.440 | 0.78-1.12 |
| Access to stoma nurse  Yes  No/do not know | 1.00  0.86 | -  -1.30 | -  **0.194** | -  0.68-1.08 | 1.08 | 0.19 | 0.851 | 0.47;2.53 |
| Clavien-Dindo  No complications  I-II  III-IV | 1.00  1.35  1.71 | -  0.84  1.20 | -  0.398  **0.229** | -  0.67-2.71  0.71-4.09 | -  1.58  2.05 | -  1.12  1.44 | -  0.264  0.149 | -  0.71-3.51  0.77-5.44 |
| Oncological treatment^¤^  None  Any | 1.00  0.75 | -  -0.77 | -  0.439 | -  0.35-1.56 | -  - | -  - | -  - | -  - |

Table S2: Logistic regression analysis on the Swedish cohort showing correlations to the anchor question

| The NETHERLANDS | Univariable regression | | | | Multivariable regression | | | |
| --- | --- | --- | --- | --- | --- | --- | --- | --- |
| Logistic regression | OR | Z | P | 95% CI | OR | Z | P | 95% CI |
| Age | 1.00 | 0.02 | 0.98 | 0.97-1.03 | - | - | - | - |
| Sex | 0.79 | -0.57 | 0.566 | 0.36-1.74 | - | - | - | - |
| BMI | 1.02 | 0.60 | 0.548 | 0.95-1.11 | - | - | - | - |
| Employment  Employed  Unemployed  retired | 1.00  1.87  1.64 | -  0.75  0.87 | -  0.452  0.385 | -  1.98-5.09  0.54-4.99 | -  - | -  -  - | -  -  - | -  - |
| Marital status  Married  Single/widowed | 1.00  1.55 | -  1.06 | -  0.290 | -  0.69-3.46 | -  - | -  - | -  - | -  - |
| Education*  None  Short  long | -  0.73  1 | -  -0.63  - | -  0.527  - | -  0.28-1.91  - | -  -  - | -  -  - | -  -  - | -  -  - |
| Financially burdened | 1.94 | 1.15 | 0.252 | 0.63-6.00 | - | - | - | - |
| Colostomy Impact group  Minor CI  Major CI | 1.00  2.08 | -  1.81 | -  **0.070** | -  0.94-4.63 | 1.00  2.08 | -  1.81 | -  0.070 | -  0.94-4.63 |
| Time since stoma creation | .99 | -0.08 | 0.937 | 0.85-1.16 | - | - | - | - |
| Access to stoma nurse  Yes  No/do not know | 1.00  1.71 | -  0.90 | -  0.366 | -  0.53-5.51 | - | - | - | - |
| Clavien-Dindo  No complications  I-II  III-IV | 1.00  0.56  1.48 | -  -1.01  0.76 | -  0.314  0.444 | -  0.190-1.72  0.54-4.03 | -  -  - | -  -  - | -  -  - | -  -  - |
| Oncological treatment^¤^  None  Any | 1.00  0.57 | -  0-1.00 | -  0.317 | -  0.19-1.71 | -  - | -  - | -  - | -  - |

Table S3: Logistic regression analysis on the Dutch cohort showing correlations to the anchor question

| SPAIN | Univariable regression | | | | Multivariable regression | | | |
| --- | --- | --- | --- | --- | --- | --- | --- | --- |
| Logistic regression | OR | Z | P | 95% CI | OR | Z | P | 95% CI |
| Age | .95 | -2.92 | **0.003** | 0.92- 0.98 | 0.95 | -2.6 | **0.008** | 0.92-0.99 |
| Sex | 1.04 | 0.11 | 0.910 | 0.53-2.03 | - | - | - | - |
| BMI | 1.01 | 0.57 | 0.567 | 0.96- 1.08 | - | - | - | - |
| Employment  Employed  Unemployed  retired | 1.00  1.79  0.83 | -  0.57  -0.21 | -  0.570  0.831 | -  0.24-13.21  0.15-4.44 | -  -  - | -  -  - | -  -  - | -  - |
| Marital status  Married  Single/widowed | 1.00  1.17 | -  0.47 | -  0.638 | -  0.62-2.21 | -  - | -  - | -  - | -  - |
| Education*  None  Short  long | -  0.73  1.36 | -  -0.79  0.57 | -  0.428  0.570 | -  0.33-1.59  0.47-3.92 | -  -  - | -  -  - | -  -  - | -  -  - |
| Financially burdened | 0.94 | -0.19 | 0.851 | 0.47-1.85 | - | - | - | - |
| Colostomy Impact group  Minor CI  Major CI | 1.00  2.83 | -  3.06 | -  **0.002** | -  1.45-5.50 | -  2.71 | -  2.67 | -  **0.007** | -  1.20-5.61 |
| Time since stoma creation | .99 | -0.18 | 0.859 | 0.91- 1.09 | - | - | - | - |
| Access to stoma nurse  Yes  No/do not know | 1.00  0.39 | -  -1.98 | -  **0.047** | -  0.15-0.99 | 0.28 | -2.12 | 0.034 | 0.09-0.91 |
| Clavien-Dindo  No complications  I-II  III-IV | 1.00  1.03  2.17 | -  0.07  0.82 | -  0.948  **0.180** | -  0.47-2.23  0.76-4.18 | -  1.09  2.09 | -  0.19  1.48 | -  0.846  0.140 | -  0.54-1.16  0.79-5.54 |
| Oncological treatment^¤^  None  Any | 1.00  2.11 | -  1.45 | -  **0.146** | -  0.77-5.82 | -  1.47 | -  0.67 | -  0.502 | -  0.48-4.47 |

Table S4: Logistic regression analysis on the Spanish cohort showing correlations to the anchor question
